# Headland, on the Action of Medicines

**Published:** 1856-04

**Authors:** 


*On the action of Medicines in the System.* By F. W. Headland, M. B., B. A., F. L. S., &c., &c. Published by Lindsay & Blackiston, Phila.

If our advice was required in the selection of good standard recent works, among others, which we could enumerate, we would most assuredly include the above most valuable treatise. It affords us much pleasure always to receive contributions to medical literature, and to speak of them to our readers, but that pleasure was increased upon the reception of this work, and as largely enhanced when recommending it to our friends. We have no fears that those who are struggling to keep pace with the medical literature of the day, will be dissatisfied, upon a perusal of it; on the contrary, it will be regarded by all as absolutely a treasure.

There is very much now afloat not worthy of commendation, and we are determined to take no notice of such. The possession of the work is not, in such cases, sufficient to warrant, for when we call the attention of our readers to any production, it will be for *their* benefit more particularly. We need in this State, a depot for the new publications, in order that those who reside back in the interior may be able to secure them early. Very many in our State would secure many of these works as fast as published, but the difficulties in procuring them are such, that they are compelled to wait until our booksellers shall have heard of their value, when they purchase them. All other standard works are kept by them in our city, but our medical men are impatient when they hear of a new and valuable work, until they can procure it.

